# The impact of Covid-19 on inter-organizational coordination in Swedish eldercare: a mixed methods study

**DOI:** 10.1186/s12913-025-12576-1

**Published:** 2025-03-21

**Authors:** Sébastien Lindhagen, Anton Modigh, Ulrika Winblad

**Affiliations:** 1 Health Services Research, Department of Public Health and Caring Sciences, Uppsala University, Uppsala, Sweden; 2 Department of Government, Uppsala University, Uppsala, Sweden

**Keywords:** Inter-organizational, Medical care coordination, Eldercare, Crisis, Covid-19

## Abstract

**Background:**

In Sweden, healthcare provision for the frail elderly entails coordination between municipalities and regions. Despite formal agreements, deficiencies persist in achieving practical coordination, leading to adverse effects on patients and increased costs. The Covid-19 pandemic further strained the health- and social care system, exposing shortcomings in eldercare coordination. This paper explores the impact of crises on inter-organizational coordination between long-term organizational collaborators, operationalized through medical care coordination in Swedish nursing homes during the Covid-19 pandemic.

**Methods:**

The study examines coordination between regionally employed physicians and municipal nursing home nurses through a mixed methods approach. A survey was sent to regional physicians and municipal nurses working in eldercare, as well as managers at both nursing homes and healthcare centers. A total of 170 responded to the survey, and 20 participants took part in a subsequent follow-up interview.

**Results:**

Findings indicate that medical care coordination was perceived to have functioned relatively well during the pandemic and even to have improved afterward. Key factors contributing to this outcome include the adoption of innovative solutions, such as digital technologies, to address both staff shortages and increased demand brought on by the crisis. Trust and shared cultural values among staff fostered collaboration, while personal engagement became crucial when compatibility was lacking. The respondents also highlighted improved communication channels and enhanced coordination as a means to combat uncertainties during the crisis.

**Conclusions:**

The perception of well-functioning crisis coordination among the respondents contrasts with more critical views in general society. This discrepancy might be attributed to different expectations during crises; healthcare professionals adhere to specified standards, values, and beliefs within their specialized cultures. Healthcare professionals might therefore have a more nuanced perception of what they believe constitutes good medical care coordination. The contributions of this study include integrating the crisis management literature with inter-organizational coordination in healthcare. The approach provides new insights to clarify the impact of crises on medical care coordination and identify important factors regarding inter-organizational coordination during crises.

**Supplementary Information:**

The online version contains supplementary material available at 10.1186/s12913-025-12576-1.

## Introduction

The Covid-19 pandemic has had a profound impact on society, with older adults being particularly vulnerable [1, 2]. The pandemic’s widespread impact has exposed the fragility of healthcare systems globally, testing their functionality and capacity [3]. Amid this global crisis, the crucial role of organizational coordination in facilitating healthcare during crises became particularly apparent [4, 5]. The pandemic has underscored the necessity of coordination between health- and social services in order to deliver high-quality care to the vulnerable elderly population affected by Covid-19 [6, 7]. Innovative approaches, such as the deployment of mobile medical teams for home-based care or provision of enhanced medical care within nursing facilities, have emerged as important strategies to this end during the prolonged crisis.

Despite extensive research underscoring the importance of coordination and resilience in healthcare systems during the Covid-19 pandemic [3, 5, 8], a comprehensive theoretical framework elucidating this relationship remains absent. Insights from crisis management literature offers a promising avenue to address this gap, as it explores how organizations navigate disruptive and unpredictable events such as the Covid-19 crisis [9]. 

This event posed a threat to the healthcare organization and its stakeholders, prompting a focused exploration of strategies to mitigate potential harm. Within this literature coordination is considered as both a problem and a solution [10]. Aldrich [11] suggests that major crises can benefit coordination by creating a sense of urgency and pushing organizations to work together. While there are potential advantages to coordination in times of crisis, it remains clear that success in coordination still poses a great challenge. The crisis requires a government response with new rules, structures, and expectations, which can conflict with the current system. Despite the generally well-intentioned nature of most coordination efforts, they risk becoming counterproductive if actors fail to coordinate effectively. Often, coordination failure during crisis poses a threat to an already dire situation [10]. 

Studies investigating coordination during crises typically distinguish between inter-organizational and intra-organizational coordination. Studies on inter-organizational coordination have mainly focused on coordination between state actors, civil society, humanitarian organizations, and emergency agencies who are compelled to work together, particularly during natural disasters such as earthquakes or tsunamis [11, 12]. The intra-organizational literature, on the other hand, often focuses on the coordination that emerges within individual entities, such as the healthcare sector, during times of crises [13]. While the literature in both these fields is extensive, neither adequately addresses the impact of crises on inter-organizational coordination between organizations that have long been working closely together, even *before* a crisis, in complex policy areas such as health- and social care.

In Sweden, healthcare services for the frail elderly are delivered by both municipalities and regions, heightening the need for effective coordination of care. The regions serve as the main provider of healthcare, while municipalities are responsible for social care including nursing homes and home-based care [14]. Over the past, there has been recurring criticism of coordination in Swedish eldercare [15]. Despite a long history of formal coordination agreements and plans, there are still shortcomings in the practical coordination of services for elderly patients between the regions and the municipalities, resulting in significant suffering and adverse medical effects for patients, as well as high costs to the system [16–18]. Prominent barriers to successful coordination in Swedish eldercare include staff shortages, a lack of incentives to promote coordination, and challenges for municipalities to involve regional physicians [14]. 

The Covid-19 pandemic has placed significant stress on the Swedish health and social care system. The eldercare system in particular has been criticized for not protecting older residents to the necessary extent during the pandemic [17]. During the pandemic the media frequently portrayed shortcomings in eldercare and commonly depicted scenarios of insufficient actions taken to reduce spread of disease or even breakdowns of eldercare wards [19, 20]. Research also points to a deterioration in care quality for elderly residents in Swedish nursing homes and hospitals during the pandemic [21–23]. The perception of failure, during the Covid-19 pandemic, is further exacerbated by the national Covid-19 Commission, which has identified substantial problems with coordination during the pandemic, focusing in particular on the fragmented organization and unclear responsibilities within eldercare [15, 24]. Although the general perception of eldercare in Sweden during the Covid-19 pandemic was that of failure, exactly how coordination in Swedish eldercare failed, and how it was impacted by the pandemic, remains unclear.

This study aims to investigate how the Covid-19 pandemic impacted the coordination between health and social care providers in eldercare. It seeks to uncover both the facilitators that aided coordination and the barriers that hindered it, in the particular context of a prolonged crisis. The study focuses on the impact of coordination and its facilitators and barriers during Covid-19, and the influence on coordination post-pandemic.

The paper is structured as follows. First, we review the literature on coordination during crises and highlight the different strands of research. We then formulate the case of medical care coordination in Swedish nursing homes during Covid-19 as an example of coordination in eldercare during crises. Thereafter, the interview methods used to understand the perception of the coordination during crisis and how it evolved are presented. Utilizing the framework from Moshtari & Gonçalves [12] on coordination during crisis, we group the findings from the interviews to identify barriers to- and facilitators for coordination during crises. Finally, we present the results, which suggest that coordination in eldercare occurs mainly in three dimensions, and that, in contrast with the general perception of eldercare, respondents believed medical care coordination to function better than expected.

### Coordination in crisis management

Pearson and Clair [25] defined an organizational crisis as “a low probability, high-impact event that threatens the viability of the organization and is characterized by ambiguity of cause, effect and means of resolution as well as by a belief that decisions must be made swiftly”. Disaster is a concept closely related to crisis and will be used interchangeably in this study, as both concepts capture a sudden event that disrupts an entire system [26]. The Covid-19 pandemic can, in line with this definition, be described as a major organizational crisis which tested all healthcare systems [5, 8]. Covid-19 was declared a *Public Health Emergency of International Concern* on January 30, 2020, and remained so until May 5, 2023 [27, 28]. The pandemic impacted society with varying intensity and in multiple waves around the world, but a decrease in disease transmission has been observed since the vaccines became available in late 2020 [29]. 

During the Covid-19 pandemic, numerous measures were implemented to maintain high-quality healthcare services. A commonly used concept in crisis management is resilience, which can be defined as the ability to absorb, adapt, and transform to cope with shocks to ensure sustained performance [8]. Factors associated with the resilience of an organization in a crisis are material and human resources, leadership in planning and managing information, and coordination [4]. Barasa [4] argue, however, that coordination can be equally, or even more important, than resources. This is, in part, because successful coordination facilitates the mobilization of both material resources between the organizations that collaborate, as well as utilizes the social networks amongst the organizations to increase the transfer of knowledge. Boin and Bynander [10] raise questions about coordination during crisis and suggests that when crisis response fails, coordination is often identified as both the underlying problem and the recommended solution. However, apart from some positive examples, coordination during crises tends to falter, resulting in inadequate crisis response [10, 11, 30]. 

The breakdown of effective coordination during a crisis can be attributed to a multitude of contributing factors. Aldrich [11], for instance, suggests that minor crises might be less successful in achieving effective coordination compared to major crises since they do not bring about enough urgency among key actors to mobilize coordination efforts. Boin & Bynander [10] partially support this claim and acknowledge that a major disaster generates a willingness among all actors to assist and coordinate services. However, they emphasize that even though urgency can drive coordination, a major crisis often results in a capacity deficit, such as of staff or certain types of organizations, that needs to be replenished by actors who may never have coordinated their actions before, making it challenging to achieve successful coordination. With the challenge of a new decision-making structure, new actors coordinating and new tasks to coordinate, there is a significant risk of fragmentation and distrust among coordinating organizations, which can result in coordination failure [10]. 

While Aldrich [11] and Boin & Bynander [10] provide different explanations for when coordination fails or succeeds, additional research on coordination in crisis management delves into a spectrum of barriers and facilitators to both intra- or inter-organizational coordination. The literature specifically focusing on intra-organizational coordination, which refers to coordination within a single organization, investigates how different parts, such as departments and specialized services within a complex organization, coordinate their actions during a crisis. Yousefian [13] conducted a systematic review developing a framework of factors that affect intra-organizational coordination in the healthcare sector during crises. However, their framework does not encompass the barriers and facilitators that impact coordination between *different* organizations that already collaborated *before* the crisis, as exemplified by health and social care for the frail elderly population. In this regard, existing studies concerning inter-organizational coordination often center on scenarios where previously unacquainted organizations are compelled to collaborate due to exigent crises. However, these studies pose a challenge as they are conducted on actors who lack prior familiarity with one another. An illustrative example of this type of coordination occurred during the 9/11 terror attacks when the U.S. Coast Guard and private boats effectively coordinated the evacuation of 500 000 people in a single afternoon [10]. 

An alternative approach is to select a non-crisis healthcare-focused coordination framework. Auschra [31] created a framework for inter-organizational healthcare coordination, categorizing barriers from clinical to administrative and political levels. However, in this study we are primarily interested in micro and meso-level coordination, i.e., coordination between health and social care providers at the “lower” levels, i.e., physicians and nurses working in nursing homes. Nevertheless, the integrated care literature has explored this level of coordination, offering a valuable starting point. Research in this field highlights key factors for successful coordination, including adequate resources, organizational culture, leadership attitudes, and engagement among clinical staff [32–34]. Studies on integrated health and social care further suggest that effective leaders should inspire collaboration, create the necessary conditions for teamwork, balance multiple perspectives, navigate power dynamics, adopt a holistic approach, commit to continuous learning, and clarify complexity [35, 36]. While these insights contribute to a broader understanding of coordination, they do not fully capture the unique contextual challenges posed by a crisis. Thus, in this paper we instead draw on Moshtari & Gonçalves [12] framework which revolves around three pivotal factors exerting influence on coordination in crisis.

While there are more appropriate frameworks for analyzing coordination in healthcare [31, 37], the impact of the Covid-19 crisis has emphasized the importance of relying on a more crisis-oriented framework, such as the one proposed by Moshtari & Gonçalves [12]. Although primarily designed for humanitarian organizations, their framework can contribute to the healthcare literature by delving into crisis-specific barriers and facilitators to coordination. However, a limitation of this framework is that it has primarily been developed based on crises caused by natural disasters, such as earthquakes or tsunamis. In contrast, the Covid-19 pandemic represents a prolonged crisis, requiring sustained coordination over an extended period with varying levels of intensity. Nevertheless, it is crucial to recognize the significant impact the pandemic had on the healthcare sector, which, in many ways, parallels the challenges posed by other large-scale disasters.

Considering the argument for a crises-oriented framework, another viable alternative could have been to select a framework from the specific crisis management literature. Numerous studies have tried to capture how to best manage a crisis and listed important factors such as crisis preparedness, communication, and learning [38–40]. However, it is important to note that this literature primarily focuses on crisis management in a general context, which diverges significantly from the context of inter-organizational coordination in healthcare. By adopting a crises-oriented framework specifically designed for inter-organizational coordination, such as the one by Moshtari & Gonçalves [12], our hope is to identify a framework that best suits our specific case. We have, however, adjusted this framework somewhat to better suit our case and to make it practically applicable, see Table 3 (adjusted framework) and Appendix 1 (original framework).

### Conceptual framework for inter-organizational coordination in crises

**Fig. 1:**
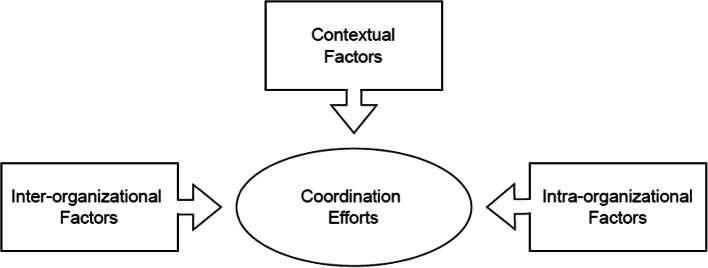
Presents factors that influence coordination in the conceptual framework

Moshtari & Gonçalves [12] present a conceptual framework based on a meta-analysis of 28 articles, aiming to categorize facilitators and barriers to inter-organizational coordination during crises. The framework is comprised of three types of factors: ‘contextual’ factors, ‘inter-organizational’ factors, and ‘intra-organizational’ factors, with coordination efforts positioned at its core (see Fig. 1). This combination helps to better understand the facilitators and barriers that drive coordination. By adopting a more comprehensive perspective on crisis coordination, a more profound understanding of its mechanisms and dynamics is revealed. Unlike many other frameworks that concentrate on just one or two aspects of coordination, such as management approaches or how organizations interact, this framework takes a more comprehensive approach by also including contextual factors.

In the Moshtari & Gonçalve [12] framework, the contextual factors are determined by broader conditions, such as supply and demand for coordination actions or the timing of the crisis. Inter-organizational factors highlight the interaction among the coordinating parties, such as the relationship between staff and the formal arrangements in the form of responsibilities or guidelines. Lastly, intra-organizational factors encompass aspects within each individual organization and reveal available resources and staff capabilities. The conceptual framework as presented by Moshtari & Gonçalves [12] is found in Appendix 1.

### Eldercare in Sweden: a case of established inter-organizational coordination

In Sweden, responsibility for eldercare services is divided between the regions (healthcare) and the municipalities (social care). The 21 regions have the primary responsibility of providing both inpatient and outpatient healthcare services to the public, including older people. This could include services at hospitals or services at so-called primary healthcare centers. On the other hand, the 290 municipalities are responsible for social care which includes nursing home care and home-based care [41]. This includes providing services and care that older adults cannot do themselves, such as personal hygiene, dressing, and cooking. Furthermore, the municipalities are legally obligated to provide services, such as providing medications, wound dressing, checking vital signs, and managing catheters. However, municipalities are not allowed to employ their own physicians; instead, they must reach agreements with the regions regarding the medical services provided by physicians [14]. 

The formal agreements between the regions and the municipalities specify that the regional physicians are allocated time each week to visit nursing homes within the municipality and provide medical care to the residents [14]. The coordination between the two organizations primarily involving the regional physicians and the nursing home nurses is referred to in this study as “medical care coordination”.

These staff groups then meet and coordinate medical care for the patients. Physicians can, among other things, help with referrals to hospitals and medical prescriptions, while the nurses have an overall knowledge about the patient. This division of responsibilities, where physicians belong to the region and nurses to the municipality, makes everyday care difficult and relies on well-functioning coordination [42]. The need for coordination has led to persistent and ongoing collaborative efforts among municipalities and regions at a higher, more administrative level [14]. This has resulted in the creation of formal coordination agreements and communication channels. For instance, all regions have entered into agreements with their constituent municipalities to ensure a certain number of physician visits per week at nursing homes. The Swedish eldercare system can thus be regarded as a representative case of well-established formalized inter-organizational coordination in the field of eldercare.

In the Swedish eldercare system, where established coordination structures already exist, the division of responsibilities during a potential crisis is governed by the so-called ‘responsibility principle’ (*ansvarsprincipen*) [43]. According to this principle, any organization responsible for a specific activity under normal conditions retains that responsibility during a crisis. In the context of eldercare coordination, social care remains the responsibility of the municipality, while healthcare falls under the jurisdiction of the region.

Despite the presence of established channels and coordination agreements, eldercare coordination in Sweden continues to face persistent difficulties. Several government reports indicate that inadequate coordination in eldercare has had negative effect on patients, resulting in increased hospital readmissions and a lack of person-centered care [16–18]. The issue of coordination was particularly critical during the pandemic, as criticism arose that some physicians refused to visit nursing homes due to fear of infection. For instance, in Swall [23], 33% of the responding registered nurses, working in eldercare, stated that physicians were seldom or never present for bedtime treatment or assessment during the Covid-19 crisis. Additionally, there were concerns that patients were not being referred to hospitals as necessary.

Eldercare coordination occurs at the micro, meso, and macro-levels [44]. At the micro-level, eldercare coordination encompasses daily operations, where physicians and nurses address the care and nursing needs of patients in nursing homes. Meanwhile, the meso-level coordination involves managers from nursing homes and healthcare centers working together to facilitate inter-organizational coordination between the organizations. Finally, the macro-level entails municipalities and regions collaborating to develop more general agreements for coordinated action in eldercare.

Barriers for coordination in eldercare can encompass different things, such as legal considerations (different laws to use as a basis for decisions between the regions and municipalities), organizational factors (involvement of two different organizations) and cultural factors (involvement of different professional groups). Previous reports [15] show considerable problems with coordination in Swedish eldercare. Among other things, barriers for coordination stem from staff shortages among all professional groups including physicians, nurses, and assistant nurses [15]. This scarcity of staff poses challenges in establishing stable coordination systems. Another barrier is the lack of financial incentives for organizations to engage in coordination, leading to a lack of interest in coordination efforts for the benefit of the patient [14]. Both of these barriers hinder the involvement of the regional physicians crucial for achieving successful medical care coordination given that municipalities are unable to employ their own physicians. Additional criticism has been directed at medical care coordination in eldercare, with specific concerns about physicians spending insufficient time with residents in nursing homes [14]. Sweden’s Health and Social Care Inspectorate [45] has also raised concerns about the country’s medical care coordination efforts, highlighting issues with follow-up on measures, shortcomings in information exchange between agencies, and an unclear allocation of responsibilities.

## Methods

### Design

The study adopted a mixed-methods approach, utilizing data from both surveys and interviews [46]. The first phase, initiated in June 2022, involved a cross-sectional survey. The second phase, launched in September 2022, consisted of qualitative interviews with participants who expressed willingness during the first phase. Data from the survey was analyzed first, and the insights gained informed the development of the interview guide.

### Setting

**Fig. 2:**
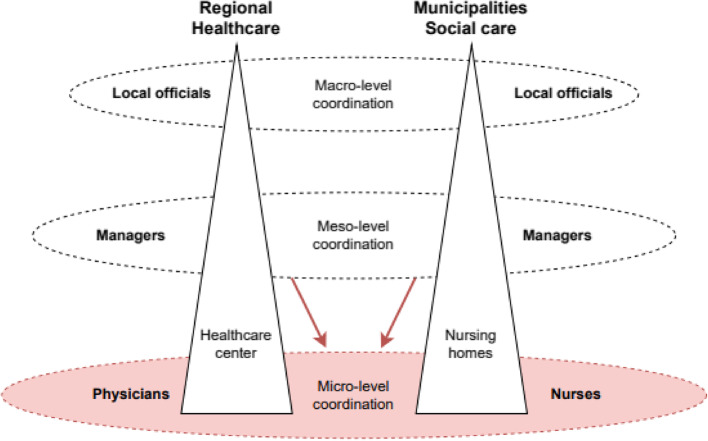
Illustrates medical care coordination in the different tiers of regions and municipalities

To investigate coordination within eldercare during a crisis, the study focused on the case of medical care coordination in eldercare during the Covid-19 pandemic, specifically the coordination between regionally employed physicians (healthcare) and nurses working within municipal nursing homes (social care). As illustrated in Fig. 2, coordination within Swedish eldercare takes place at different levels and between different organizations. However, the aim of this study was mainly to understand the ‘medical care coordination’ between physicians and nurses, during the Covid-19 pandemic, on the micro level. This coordination could include discussions about individual patients, such as decisions about palliative care, referrals to other caregivers, or decisions regarding medical intervention, such as new medications. Some of the results include issues that are more at the meso level. In these cases, the discussion revolves around matters of a more administrative nature, such as how to implement guidelines or agreements and create favorable conditions for medical care coordination at the micro level.

### Quantitative study: a survey of professionals and managers

A cross-sectional survey was conducted with the aim of capturing how the Covid-19 pandemic impacted the coordination between physicians (healthcare) and nurses (social care) during the pandemic, and also the perceived state of coordination post-pandemic. A sample of three regions was chosen based on geographical location, size, and similar median Covid-19 mortality rates [47]. The survey was sent to regional physicians and municipal nurses working in eldercare, as well as managers at both nursing homes and healthcare centers, between June and October of 2022. The invitation to the survey was carried out in two phases. In the first phase, respondents whose contact information was available from either the regional or the municipal websites, mostly managers of nursing homes and healthcare centers, were invited.

The second phase involved identifying respondents whose contact information was not available online, primarily nurses and physicians. In cases where contact information was unavailable, respondents were reached through an intermediary person within the region or municipality who assisted in sending out the invitation. When using an intermediary person, the researchers did not receive information about the number of invitations distributed. In the first phase, 427 respondents were invited to participate in the survey. For the second phase, no information about the number of respondents invited is available.

The survey questions were specifically developed for this study, drawing on prior knowledge in the field and further developed in a structured dialogue with researchers and experts in the field. Following the initial development, a nurse and a physician with prior experience of coordination within eldercare were invited to pre-test the questionnaire. Revisions were then made based on their feedback. Finally, the survey was tested and refined after an academic seminar with researchers and several experts in the field.

The survey comprised 18 items with various response options such as yes or no choices, Likert scales, and open-ended responses. The items were structured as follows: The first set of items investigated respondents’ roles and experiences of current care coordination. This was followed by 11 items examining common tasks within medical care coordination comparing the year 2019 and 2022 cross sectionally. The items were selected to capture specific conditions during pre- and post-pandemic levels of coordination. Questions comparing the year 2019 and 2022 were based on statements such as “*Medical care coordination of eldercare in emergency situations works well*” and “*It is easy for physicians and nurses in eldercare to get in touch with each other in their daily work*”. Respondents graded the statements on a 7-point Likert scale with one being “*Strongly disagree*” and seven being “*Strongly agree*”; there was also the option to answer, “*don’t know*”. In addition, respondents were questioned on medical care coordination during the crisis, evaluating if the crisis improved, deteriorated, or had no impact on coordination, using response options “*It has generally improved*”, “*It has generally deteriorated*”, “*It has both improved and deteriorated*”, and “*It has not been affected at all*,* but works in the same way as before*”. For a detailed list of questions and response options see Appendix 2. Respondents were informed that the study was approved by the Swedish Ethical Review Authority (dnr. 2022-01791−01).

A Wilcoxon signed-rank test was utilized to determine whether there was a significant difference in the median values between the years 2019 and 2022. Descriptive statistics were calculated consisting of median and mean values, as well as the standard deviations and mean differences.

### Qualitative study: interviews with professionals and managers

The aim of the qualitative analysis was to discern barriers and facilitators regarding medical care coordination in eldercare during the Covid-19 pandemic. To identify potential interview participants, the survey included a question asking respondents if they were willing to take part in a follow-up interview.

The interview guide was developed with the help of experts in the field of health services research. It consisted of targeted questions and subsequent follow-up questions and was structured around four sections. First, participants were asked background questions and about their role in medical care coordination. The second section involved questions about coordination between organizations, including the current state of medical care coordination and what factors contributed to the success/failure of coordination. Participants were then asked about the changes to coordination in post-pandemic, including what factors contributed to the improvement/deterioration of coordination. The last section included questions about sustainable improvements, what contributes/hinder these improvements over time. The goal of the final section was to encourage participants to envision factors for sustainable improvements without needing to connect them specifically to their organization. To view the interview guide, see Appendix 3. Two pilot interviews were conducted: one with a manager and one with a nurse. Following these interviews, some questions were revised to better align with the aim of the study. Both pilot interviews were subsequently included in the study.

A total of 35 participants expressed interest in an interview and received an email invitation. Among the participants there was a spread in characteristics of profession and geographical region. Interview requests were sent out between September and December of 2022, with interviews conducted simultaneously as the distribution of invitations was done. Semi-structured interviews were conducted via online platform Zoom and had a duration of 18 to 37 min (on average 26 min per interview). The interviews were pseudonymized and transcribed using a semi-verbatim approach. Two research assistants, AM and SL, conducted the interviews under the supervision of UW. All researchers (AM, SL, UW) were members of the health services research group, possessing knowledge of organizations and outcomes of healthcare systems from different perspectives. Before the interviews, participants received information about the topic of the interview and the researcher conducting the interview.

The interviews were analyzed using a qualitative deductive content analysis approach [48]. In the initial phase of the deductive content analysis, a categorization matrix was developed using a deductive approach based on the conceptual framework by Moshtari & Gonçalves [12]. Data were classified independently by AM and SL according to the matrix, aligning with the pre-existing factors and categories within the framework (see Appendix 1 for original framework). During the coding process, AM and SL conducted a preliminary test of the first interview, to assess the alignment of their codes before proceeding with the remaining interviews. While the framework was extensive and encompassed numerous elements, the subsequent phase employed an unconstrained matrix approach. This involved adjusting and refining categories and subcategories to suit the unique context of medical care coordination within an eldercare setting. The process of adjusting and refining categories and subcategories was employed jointly by AM and SL with supervision from UW. The unconstrained matrix approach utilizes the process of adjusting and refining categories following the principle of inductive content analysis [48]. During the analysis some categories, such as ‘use of funds’ and ‘inter-organizational competition,’ were removed, as they did not align with the case of an eldercare setting. Additionally, the category ‘environmental unpredictability’ was excluded because it fell outside the scope of participants´ expertise, as it involves information on a more macro-level, with subcategories such as ‘political environment’ and ‘timing of the crisis’. The two categories ‘operational compatibility’ and ‘strategic compatibility’ were merged into one category, as most subcategories of ‘operational compatibility’ aligned with the concept of ‘shared cultural values’ within ‘strategic compatibility’. Meanwhile, the categories ‘unclear benefits of collaboration’ and ‘collaboration capabilities’ were renamed to more accurately reflect the subcategories within them.

### Non-response analysis

To address the low survey response rate, we conducted a non-response analysis. This analysis included two key components: first, we conducted telephone interviews with randomly selected non-respondents whose work-related information was available online. During these interviews, we emphasized that their feedback would be used to improve further survey invitations and clarified that this was not a request for them to revisit the initial invitation. Respondents cited three primary reasons for non-response. They mentioned either missing the survey, lacking the time to complete it at the time of its distribution, or believing that the survey should have been directed to another individual.

Second, we scrutinized the email addresses used for survey mailings. Our findings revealed that 12–22% of the email addresses used for survey mailings were categorized as ‘invalid’, indicating that it is a challenge to find correct contact information in the target population. Results from the non-response analysis imply that the actual response rate was probably higher than the calculated rate.

## Results

In total, 170 respondents participated in the survey. Among the collected responses, 48% were from nurses, 23% from physicians, 22% from nursing homes manages, and 6% from healthcare center managers. Within the group receiving a personalized invitation, 81 (19%) of the participants responded. For cases where an intermediary person assisted in distributing the invitations, no information about the number of invitations sent out was available, but 89 participants responded to the survey. Of the 35 respondents invited to participate in an interview, 20 individuals from the four professions accepted, see Table 1.

**Table 1:** Presents the characteristics and total number of participants

Roles	Participants interviewed	Respondents surveyed
Nurses	6	82
Managers of nursing homes	7	38
Physicians	5	39
Managers of healthcare centers	2	11
Total	20	170

### Quantitative survey results: the impact of Covid-19 on coordination

The responses received from the survey indicate that medical care coordination was perceived to work quite well post-pandemic. Specifically, 86% of respondents reported medical care coordination as functioning moderately to very well, while only 13% reported poor or very poor performance. There was no discernable difference among the regions and only a small difference among the different respondent groups, so results will hereafter be presented as one group. When examining the impact of the pandemic on medical care coordination, 48% of the respondents reported no discernible effect, while 7% reported deterioration, 13% reported improvements, and 13% of respondents reported both improvements and deterioration.

The survey examined statements about common tasks within medical care coordination comparing the year 2019 and 2022, resulting in mean scores ranging from 4.57 to 6.19 in 2019 and 4.8 to 6.32 in 2022. The time allocated for medical care coordination by physicians in practice received the lowest ranking, while good relations between physicians and nurses garnered the highest ranking. The overall standard deviation was 1.776, with a range from 1.298 to 2.013 (see Appendix 4 for descriptives statistics). The comparison of common tasks between the years 2019 to 2022 found a slight positive change in most of the coordinating tasks over time, but there was only a statistically significant difference in two items: “*Clarity of agreements and documents after the crisis*”, and “*Accessibility of transferring of information”* (see Table 2). The analysis of the survey data thus found no evidence of a change in coordination tasks between 2019 and 2022 except for in communication and documentation. To summarize, the survey illustrates that the pandemic did not seem to significantly alter how medical care coordination was perceived to function once the crisis had eased, with the exception of documentation and information transfer.

**Table 2:** Presents the results from a Wilcoxon signed-rank text, comparing the year 2019 and 2022

Questions*	Mean difference	Wilcoxon signed-rank test *p*-value
Agreements or other documents are clear.	0,026	0.003**
Emergency situations work well.	−0,03	0.206
Time allocated by contract corresponds to the needs.	0,011	0.455
Time allocated in practice corresponds to the needs.	0,002	0.252
The staffing of nurses is sufficient for coordination.	0,006	0.769
Same physician who provides medical services.	−0,005	0.810
Same nurse who interacts with the physician.	0,132	0.706
Physicians see patients to a sufficient extent.	0,038	0.404
Physicians and nurses getting in contact in daily work.	0,029	0.052
Transfer of information works well.	0,032	0.017**
Good relationship between physicians and nurses.	0,132	0.137

* Simplified questions. For the original survey questions, see Appendix 2

** = *p*<0.05 is considered significant

### Qualitative interview results: facilitators and barriers in coordination during the Covid-19 pandemic

The results from the survey indicate that medical care coordination was perceived to function quite well overall, with a majority of respondents rating it as working moderately to very well. The pandemic does not seem to have been detrimental to coordination, but rather improved two aspects: documentation and communication. This rather surprising result inspired a further question: why did the pandemic not deteriorate collaboration? The qualitative part of the study aimed to address questions about the aspects of coordination that contributed to the organizations’ effective function through a prolonged crisis. Table 3 provides the results from the deductive content analysis and summarizes the data into factors, categories, and subcategories.

**Table 3:** Factors influencing medical care coordination during crisis

Contextual factors	Inter-organizational factors	Intra-organizational factors
**Supply** - Supply of coordinating actions during crises	**Compatibility** - Level of trust - Shared cultural values	**Attitudes towards coordination** - Engagement with medical care - Priorities for coordination
**Demand** - Demand for coordinating actions during crises	**Partners’ power dynamic** - Hierarchies between the parties	**Available resources** - Staffing levels - Internal knowledge - Workload
	**The coordination processes** - Roles and responsibilities - Routines and guidelines - Communication	**Management and organization** - Supportive leadership - Bottom-up management

### Contextual factors

The contextual factors highlight the setting in which medical care coordination in eldercare operates. These factors primarily consist of elements that contribute to the crisis, including uncertainties about the required extent of medical care for residents and the capacity of various organizations to deliver said medical care, i.e., supply and demand factors.

#### Supply

The supply category was defined by the amount of medical care that either of the organizations could provide. In the Swedish context, the allocation of staff and services is predetermined based on each organization’s budget– that is, the budgets of the regions and municipalities. The provision of services to the residents is then managed by agreements between the region and municipality, outlining the number of allocated hours that physicians can spend in the nursing homes. According to the participants, during the prolonged crisis centralized management of supply could lead to a mismatch with actual need creating a barrier for coordination. Supply shortages would frequently emerge due to staff absences being caused by illness or virus exposure. As the supply of staff and services was reduced, participants found it harder to coordinate services.

*“… too high pressure simply. So that there is no air in the system that someone can be sick or on sick leave…”* – IP 13

Despite the challenges to coordinating services during the crisis, many of the participants insisted that the organizations were able to maintain their functionality to a significant extent and even create innovative solutions for coordination, such as increased use of digital communication.

#### Demand

Demand was defined by the amount of medical care that the elderly residents needed, as evaluated from the perspective of the participants. During the crisis, as the medical care needs of the residents changed over time, participants emphasized that the demand was rarely consistently fulfilled. Participants stated that in situations where the demand could not be met, it had the potential to lead to a diminished quality of medical care for the residents. Demand for medical care was heightened due to the transmission of the virus, for which none of the organizations were prepared. Some physicians however pointed out that, from a medical perspective, the disease did not appear to be more challenging to manage than other diseases such as influenza. The strain on the organizations was primarily due to the increased demand resulting from a larger number of patients falling ill. This heightened demand overwhelmed the organizations and made it more challenging for physicians to provide timely treatment to patients in nursing homes within their allocated time.

*“Then there was more to do, simply because people got sick. A lot of medical assessments and a lot of calling on days you [physician] weren’t there too.”* – IP 7

### Inter-organizational factors

Inter-organizational factors encompass the dynamic interactions between organizations during times of crisis. This involves not only the relationships between the professions but also other arrangements that enable coordination, such as how to communicate or what routines to follow. In terms of inter-organizational factors, three distinct categories emerged.

#### Compatibility

The compatibility category pertains to the interpersonal relationship among professionals engaged in coordinating services within eldercare. In the analysis, *level of trust* was perceived as an important facilitator for coordination, creating an outlet for nurses to ask physicians for more help or physicians to better understand the patient’s illness through the nurse’s knowledge. During the crisis, higher levels of trust further established a sense of security and stability among the nurses and the physicians. Trust was frequently mentioned as crucial given that management oversight was minimal, and physicians and nurses had to rely on each other for such things as transfer of information or emergency situations. Furthermore, trust was seen as important as it mitigated the stressful situation arising from uncertainty of the crisis. The prolonged crisis also gave rise to changes in trust, as participants described an increased appreciation and understanding of each other´s roles.

*“Because we had extra work and difficulties from both sides, we learned to have a little more tolerance towards each other, to understand each other´s responsibilities and obligations a little more concretely, because it is quite concrete, and it was not before.”* – IP2

*Shared cultural values* between physicians and nurses were key to ensuring the willingness of both parties to coordinate effectively together and maintain a shared belief in their approach. During the crisis, a joint culture meant that both physicians and nurses had similar expectations to each other and the medical care they provided, which in turn increased trust. Shared cultural values also culminated in staff feeling like part of a group, which in turn strengthened them and their coordination efforts. As the prolonged nature of the crisis unfolded, some participants stated that the crisis had a positive impact on their shared cultural values, as everyone was working toward the same goal. A manager at one of the healthcare centers said:

*“I think it’s about a common view of what quality you want and where you are going… we have decided together with our municipality as well. And that we pitch in the resources to match and that they do the same.”* – IP8

#### Partners’ power dynamic

The interviews revealed a status *hierarchy between the parties* which acted as a barrier to coordination, stemming from the fact that the municipalities were not allowed to employ their own physicians. Physicians were instead contracted by the regional healthcare centers, making the municipal eldercare nurses reliant on regional physicians to provide the necessary medical care for the residents, creating a hierarchy of dependency. The lasting crisis put a further strain on the relationship, causing the nurses to be more reliant on the physicians for medical services during a period of high uncertainty. Although the perception of trust in general improved during the crisis, participants expressed frustration at the systemic level of medical care coordination, stating that the power disparity became more apparent during the crisis due to physicians being the only ones with medical expertise, which was a problem due to the increased need for medical services.

#### The coordination processes

Within the realm of the actual coordination process, participants described the setup of medical care coordination and how organizations managed and integrated various activities and tasks. Participants discussed the importance of clarity regarding *roles and responsibilities*, as it ensured that everyone knew what to expect from their counterparts. During the crisis, participants expressed having difficulties when they felt their counterparts did not fulfill their roles, leaving them with the fallout. The crisis did not lead to new arrangements in responsibilities among the actors, due to the Swedish responsibility principle (*ansvarsprincipen*), which states that areas of responsibility should not change during crises. However, some participants described a lack of predefined roles in certain circumstances, which became more apparent during the crisis. This, in turn, led to increased difficulties in coordination, as participants could not rely on their counterparts. They expressed a tendency that staff transferred patients to other organizations they deemed responsible rather than adopting a comprehensive approach to patient care.

*“… you think that the other person is responsible for it. It’s this division of responsibility… In reality, it’s more like dumping people [patients] as soon as possible*.” – IP2

Participants also highlighted *routines and guidelines* as crucial to the foundation of medical care coordination in eldercare, facilitating interaction and ensuring that both parties knew what to expect from each other. A lack of well-defined routines or guidelines created confusion about what to do and what protocols to follow during the crisis. Although routines and guidelines were viewed as important during non-crisis periods, participants often found them challenging and hard to comply with in the context of the high uncertainty brought by the crisis. The dynamic nature of the crisis led to frequent changes in routines and guidelines within a short time span. Furthermore, the introduction of numerous new routines or guidelines during the crisis had the consequence of fostering a top-down management approach. This approach restricted the staff´s ability to think creatively and take proactive control over their tasks.

*“There was no point in reading the sampling procedures on Friday if you were not working on the weekend because on Monday they would be new again. So that’s how it felt for a while… you had to be creative and then maybe it was not according to routine all the time but you tried to get the business to flow.”* – IP5

Participants also viewed *communication* as vital in order for medical expertise to be transferred between organizations. When communication faltered, it put barriers on the coordination efforts. However, as the crisis unfolded, lines of communication were perceived to have improved according to both physicians and nurses. Communication was improved by both enhancing already established channels, such as increasing telephone availability, and introducing new methods of communication through digital solutions. Some healthcare centers, for example, arranged a specific contact person for municipal eldercare staff to call when they needed assistance outside of their scheduled appointments.

*“… [we now] have a contact person at the healthcare center… they can also call there and talk to the person who can take it to the physician and so you can get faster contact with the physician.”* – IP17

### Intra-organizational factors

Factors within each individual organization exerted a significant influence on the coordination between physicians and nurses during crises. Factors included the overall attitudes within each organization, the resources allocated, and management.

#### Attitudes towards coordination

Participants highlighted personal *engagement with medical care* as important during the crisis, stating that it could motivate participants to exert extra efforts individually. Moreover, when one party was not fully engaged in their work-related tasks, it led to mistrust and worse medical care coordination. Physicians were frequently identified by the nurses as lacking engagement, which they attributed to the fact that physicians were only contracted in nursing homes to provide limited medical services and were only present on an irregular basis. During the prolonged crisis, personal engagement was seen as vital as it made organizations more resilient to unexpected changes, motivating the participants to work through tough times. Although engagement was identified as important, participants in our study did not indicate that unengaged staff became more engaged due to the crisis.

The degree to which organizations *prioritized coordination* in eldercare was believed to affect the attitudes of staff and thereby the success of coordination. During the crisis, physicians reported that when the management of their organization did not prioritize medical care coordination with the nursing homes, there was little time and opportunity to engage in this type of activity. Medical care coordination with eldercare is only one of many assignments for which the regional physicians are responsible, making it difficult to prioritize. However, some physicians pointed out that as the demand for medical care by physicians in nursing homes increased, they could exert pressure on their regional managers at healthcare centers to prioritize medical care coordination to a higher degree.

#### Available resources

The availability of intra-organizational resources affected the extent to which staff could engage in medical care coordination in the eldercare setting. The *staffing levels* within one organization were seen as pivotal for medical care coordination, and low levels of staffing or weak continuity among the physicians or nurses were identified as a barrier to coordination. During the crisis, participants stated that low staffing levels impaired the development of relationships during coordination, creating a lack of compatibility and decreasing participants’ willingness to ask their counterparts for help. Higher staffing levels meant that there was room in the system for unplanned events. The absence of staff members led to a disruption in staff continuity. As a result, it became more challenging to coordinate among involved organizations. One of the participants highlights continuity as an important aspect for coordination.

*”We’ve had this physician for a while now, so we know each other well and how to work together, so maybe that has something to do with the stability of working together…”* – IP1

Participants found that possessing *internal knowledge* about medical care coordination, including coordination procedures and patient-specific information, played a crucial role in enabling them to effectively collaborate with their counterparts. During the crisis, participants acknowledged the significance of harnessing the existing knowledge within their respective organizations to enhance their ability to coordinate effectively with the other organization. This was partially accomplished by implementing clear instructions, streamlining procedures, and developing comprehensive coordination guidelines that were also comprehensible to the counterpart actors. However, the nature of the crisis resulted in a significant increase in staff absenteeism for both nurses and physicians. The increase in staff absenteeism led to a loss of internal knowledge about coordination procedures, including information transfer and appropriate communication channels. Limited familiarity with patients and their medical histories could result in staff members overlooking vital patient information.

*”I think you can lose a lot of information if there is a turnover of both physician and nurses. It’s like starting over when a new nurse starts.”* – IP11

Participants described their *workload* as an important barrier affecting how well they could coordinate with their counterparts. During the crisis, physicians in particular stated that high workloads could create confusion as to how to prioritize, sometimes leading to medical care coordination not receiving the time needed to function efficiently. Furthermore, greater workload was a recurring theme and often created a sense of time scarcity, which in turn could culminate in stress.

#### Management and organization

A further factor affecting medical care coordination in eldercare could be linked to how the management within each organization worked. A *bottom-up* approach to management was seen as an important facilitator to coordination as it created an environment of flexibility and adaptability. During the crisis, innovations within the organizations were primarily found to be driven by the staff working closely with patients, highlighting the significance of a bottom-up approach that facilitated quicker decision-making. Furthermore, innovative ways of coordinating during the crisis came from the staff working closely together ‘at the bottom’, reducing the need for a top-down management approach.

*“… you got rid of the fixed boundaries, the decisions that take too long … now it’s this patient, how should you think?”* – IP14

Moreover, according to the participants, *supportive leadership* was viewed as central in providing direction to the staff concerning the established procedures and guidelines regarding coordination. During the crisis, managers acknowledged their involvement in clinical setting as limited, owing to the staff´s expertise in the area. Although a bottom-up approach to management was important during a crisis, lack of leadership could result in less accountability, which in turn impaired medical care coordination. During the Covid-19 crisis, inadequate leadership caused confusion among staff who struggled to comply with the varying routines and guidelines from different sources; however, this did not entail that leadership had to assume a hands-on approach in every scenario. Rather, in times of crisis, leaders’ principal function revolved around furnishing support to the staff.

## Discussion

### Principal findings

The aim of this study was to investigate the impact of the Covid-19 pandemic on eldercare coordination, and to examine the facilitators that contribute to, and barriers that undermine, coordination during a prolonged crisis, such as the pandemic. As the pandemic unfolded in Sweden, the media, government reports, research, and, subsequently, the Covid-19 report portrayed coordination in eldercare as faltering, highlighting significant deficiencies in performance [15, 19, 20, 23]. However, the findings of this study indicate that medical care coordination in nursing home care was conceived to have functioned relatively well, according to the providers themselves. Quite surprisingly, the pandemic was not perceived by healthcare professionals to have led to a deterioration of medical care coordination post-pandemic, but rather to a slight improvement in some aspects. The survey data suggested that both the clarity of documents and agreements, as well as the transfer of information between organizations, improved significantly after the crisis.

The analysis of the qualitative interviews, using the framework from Moshtari & Gonçalves [12], has the potential to shed light on this somewhat surprising result, indicating that coordination did not seem to have deteriorated – in fact, it seemed to improve - according to the survey respondents themselves. Participants explained that despite the reduction in staff and services due to illness and virus exposure, organizations largely succeeded in sustaining their collaborative efforts. An explanation for this can be attributed to the inter-organizational element of compatibility, exemplified by a heightened mutual trust among staff members and the existence of shared cultural values between the organizations. This finding aligns with previous research, which identifies poor interprofessional relationships as a major barrier to successful care coordination [34]. Participants described how a higher level of trust amongst the staff during the pandemic mitigated the stressful situation and ensured that staff could ask each other for assistance outside their regular work hours. The presence of shared cultural values was also significant as it meant that organizations felt like they were a joint group, which strengthened them during the hardships and created the feeling of working towards the same goal.

However, the interview study partially contrasted with the survey results, as some participants argued that the pandemic challenged coordination through staff absences, time scarcity, and inadequate information transfers. Some participants noted that they still lacked trust and shared cultural values with their counter-organizations. This did not necessarily imply that medical care coordination did not function, but rather that other intra-organizational factors, such as personal engagement, became more significant. For example, engaged staff exerted pressure on their managers to prioritize medical care coordination to a greater extent, which led to an increase in the time allocated to it each week.

Our results seem to align well with Aldrich [11], who suggests that urgency in a major crisis can de facto *improve* coordination. In such situations, actors often feel compelled to coordinate in order to effectively solve the difficult issues at hand [11]. This might have been the case during Covid-19 where staff introduced new, innovative communication channels to enhance coordination. The adversity of the situation also appeared to foster mutual understanding and a necessity to place trust in one another. However, the pandemic also caused intensified interdependence among coordinating actors, underscoring how the failure of one actor to fulfill their responsibilities could undermine coordination, amplify power discrepancy, and fuel frustration, particularly on the part of the municipal nurses.

An alternative explanation, not raised by the crisis-oriented theories, could be attributed to disparate expectations and understanding among the participants, compared to other groups in society, of what constitutes good and effective coordination in eldercare. Healthcare professionals frequently adhere to specified standards, values and beliefs within their specialized cultures, while other groups in society have different views of what constitutes good healthcare [49, 50]. For example, both physicians and nurses within nursing homes might possess a deeper understanding of the challenges in caring for seriously ill older adults, thus fostering a more favorable view of the coordination process than outsiders. One of the participants validated this perspective by stating that the pandemic raised societal awareness about the shortcomings of coordination within eldercare, while the staff thought they were doing what they should and could to ensure high quality care. This shift in perception might explain why staff could discern improvements within their coordination efforts while the public did not.

Furthermore, the positive perception among the professionals towards medical care coordination may also be attributed to an increased sense of empowerment among the working staff during the pandemic. This is consistent with previous research that has demonstrated the importance of clinical staff engagement in successful coordination [32]. The sense of control could be explained by the responsibility principle in Sweden, stating that responsibilities do not shift during a crisis. The responsibility principle implies the absence of a central crisis organization dedicated to handling the pandemic and furthermore grants the management the overarching responsibility. Our findings suggest that the healthcare and social care management responded to this responsibility by granting autonomy to the healthcare professionals themselves to manage the crisis. This is consistent with other results from the Swedish healthcare sector during Covid-19, indicating that collegiality and peer learning prevailed over reliance on routines and regulations [51]. This further aligns with research on integrated care suggesting that managers granting autonomy to staff foster a more favorable depiction of coordination efforts [35, 52]. 

However, our findings differ somewhat from government reports and studies suggesting that coordination failed during the Covid-19 pandemic in Sweden [15, 22, 23]. One possible explanation for this discrepancy is that our survey was conducted after the pandemic, whereas the referenced studies were conducted during its peak [22, 23]. Additionally, our interviews were also conducted post-pandemic, which may have influenced participants’ recollections. Despite the risk of recall bias, our results indicate that medical coordination was perceived as functioning quite well post-pandemic, with the crisis ultimately driving improvements in certain parts of inter-organizational coordination. Another factor could be the professional groups included in our study. Previous research has shown that assistant nurses, who work closely with older people, tended to have a more negative view of coordination during the pandemic [22]. In contrast, the nurses and physicians in our study might have had different experiences, potentially leading to a more positive perception of coordination efforts.

Thus, the conclusions drawn from this study may primarily be applicable to crises related to pandemics or other diseases. Furthermore, the study’s scope is confined to inter-organizational coordination, within healthcare, with established coordination and where coordination mechanisms are already in place. We contend that the Swedish eldercare system serves as a challenging case for achieving successful coordination, given the inter-organizational divide between municipal social care and regional healthcare. Furthermore, we conclude that the study’s generalizability extends to contexts in which responsibilities of healthcare and social care are divided, such as in Swedish eldercare.

### Contributions and health policy implications

This study makes a novel contribution by integrating crisis management literature with inter-organizational coordination in healthcare. Firstly, crisis management theory was employed to explain the impact of Covid-19 on inter-organizational coordination. Secondly, a framework capturing facilitators and barriers to inter-organizational coordination during crises was adapted to a healthcare setting. Although the crisis framework identifies various factors that influence coordination outcomes such as supply and demand, it does not establish a causal link explaining their impact on coordination. Future research should put emphasis on identifying the factors that are particularly pivotal for coordination during crises.

The findings of this study highlight potential policy implications for achieving effective medical care coordination during crises such as the Covid-19 pandemic. The results suggest that success lies in maintaining already well-established coordination channels and prioritizing the improvement of these formalized mechanisms. To better prepare for future crises, organizations should focus on developing robust and reliable channels for information transfer, as the lack of such channels was identified as a significant barrier to effective coordination. Furthermore, the results indicate that established relationships with coordinating actors are important to create trust and a sense of shared goals during times of crisis. Therefore, organizations should continue to develop collaborative relationships even under more normal conditions.

### Study strength and limitations

A key strength of this study is its combination of quantitative survey results with in-depth interviews, providing a comprehensive understanding of the medical care coordination during and after the Covid-19 pandemic in Sweden. Another strength is the integration of crisis management literature with research on inter-organizational coordination in healthcare. The mixed method approach, coupled with our theoretical framework, offers new insights into how coordination was impacted by the pandemic.

This study has some limitations. First, the response rate of the survey was low, introducing a risk of bias influencing the results. One could argue that the unexpectedly positive views on coordination might be attributed to bias, assuming that participants with a more optimistic perspective were keener to participate in the survey compared to those with a more pessimistic view. We consider this scenario unlikely considering that all participants were assured anonymity from their peers and managers; on the contrary, the survey could be viewed as an opportunity to anonymously air grievances about the management of the pandemic. Instead, we believe that the low response rate stemmed from time constraints and an overload of surveys directed at this specific sampling group after the Covid-19 crisis. This view was confirmed when conducting the interview study, where some of the participants who had initially expressed a willingness to participate stated that they no longer wanted to participate due to time constraints. Additionally, incorrect email addresses, due to the absence of relevant contact lists and staff information from the websites of the regions and municipalities, led to some individuals receiving the survey who were not intended to be included in the sample, thus potentially decreasing the apparent response rate. This in connection with the high staff turnover probably led to a low response rate.

A second limitation concerning the participants relates to the reliability of their responses regarding how the coordination shifted before, during, and after the pandemic. Assessing the “before” and “during” phase was challenging due to its remote timeframe but since we lack insight into perceptions before the pandemic, it became necessary to ask these questions after the pandemic had mostly subsided. However, this design creates a risk of recall bias as the participants’ recollection may not accurately represent reality, which is a limitation to the study. A related limitation is the prolonged nature of the pandemic, with fluctuating intensity, making it challenging for participants to distinguish pandemic-related causes. Despite this challenge, we argue that the impact of the pandemic was so significant that it is likely to have left an impression strong enough for participants to recall the changes it brought. Furthermore, this challenge motivated our mixed-method approach, allowing us to complement the survey data with in-depth interviews to gain a more comprehensive understanding of the mechanisms influenced by the pandemic. This was further addressed by asking specific questions in the survey that encouraged respondents to try to recall in detail what happened. In the interviews, we asked open-ended questions about the impact of the pandemic, allowing participants to recount what had happened at their leisure.

Finally, a third limitation is that the selected framework was developed to describe coordination during crises among humanitarian organizations, rather than healthcare organizations that have coordinated efforts over an extended period of time. Nonetheless, we found this framework the most suitable to use for the purpose of this study in assessing facilitators and barriers. By adopting the framework to our particular context, we found it useful in providing a comprehensive overview of diverse factors influencing coordination. However, there is a potential risk that we might overlook factors associated with coordination in the healthcare field.

## Conclusion

The findings of this study indicate that healthcare professionals working in medical care coordination in eldercare perceived collaboration to function well after the Covid-19 crisis. This is a surprising result given the massive critique toward the management of Covid-19 in eldercare. The discrepancy is attributed to different expectations; the professionals adhere to specialized standards within healthcare that can foster different values and beliefs within their specialized culture. Furthermore, their experience in the field gave them a more nuanced perception of what they believe constitutes good medical care coordination. The conclusion of the study also underscores the importance of trust and shared values among the professionals working within organizations that coordinate during crises. Despite the challenges brought on by the crisis, a familiarity with the counter-organization mitigated uncertainties during stressful periods.

The contributions of this study include integrating crisis management literature with inter-organizational coordination in healthcare. The approach provides new insights to clarify the impact of crisis on medical care coordination, as well as identifying the facilitators and barriers that are important for coordination during a crisis.

## Supplementary Information

Supplementary Material 1.

Supplementary Material 2.

Supplementary Material 3.

Supplementary Material 4.

## Data Availability

No datasets were generated or analysed during the current study.
