# Supplementary material for: The impact of Covid-19 on inter-organizational coordination in Swedish eldercare: a mixed methods study
Source: BMC Health Serv Res. 2025 Mar 21;25:416. doi: 10.1186/s12913-025-12576-1 (PMC11927287; doi:10.1186/s12913-025-12576-1)
Supplement: Supplementary file 1 — Supplementary Material 1. [file 12913_2025_12576_MOESM1_ESM.docx]

**Appendix 1**. The conceptual framework, as presented by Moshtari and Gonçalves (2017).

| Contextual factors | Inter-organizational factors | Intra-organizational factors |
| --- | --- | --- |
| Environmental unpredictability   - Location and timing of disasters - Political environment - Security | **Strategic compatibility**   - Shared organizational objectives, missions, mandates - Shared cultural values - Shared language - Level of trust among organizations - Strength of sense of mutuality | **Unclear benefits of collaboration**   - Bureaucracy, transparency, accountability, flexibility - Required speed of response - Required independence and sovereignty - Risks to humanitarian identity or to humanitarian principles - Risks to own competencies - Incentives for collaboration |
| Demand   - Quantity, characteristics, and needs of affected population - Urgency of relief response | **Operational compatibility**   - Similar operational policies - Similar programming approaches, timeframes - Similar standards and techniques | **Available resources**   - Availability of resources - Adequate access to tools and technical skills Stability of team leaders and focal points - Seniority of coordinating staff members |
| Supply   - Remaining local infrastructure - Availability of local and international resources - Number and experience of involved HOs | **Interorganizational competition**   - Competition for funds - Competition for visibility and media coverage | **Collaboration capabilities**   - Propensity toward command and control focus - Management capacity and leadership style - Staff capability |
| Use of funds   - Resource availability timing - Required burn rates - Earmarked funds establish use | **Partners’ power disparity**   - Similarity in organizations’ power and resources - Symmetry between the parties - Fair distribution of benefits |  |
|  | **Coordination process**   - Mechanisms to allocate costs, benefits, risks - Collaboration performance evaluation - Performance accountability - Clear roles and responsibilities - Adoption of transparent and responsible policies - Communication |  |
