# Supplementary material for: The impact of Covid-19 on inter-organizational coordination in Swedish eldercare: a mixed methods study
Source: BMC Health Serv Res. 2025 Mar 21;25:416. doi: 10.1186/s12913-025-12576-1 (PMC11927287; doi:10.1186/s12913-025-12576-1)
Supplement: Supplementary file 2 — Supplementary Material 2. [file 12913_2025_12576_MOESM2_ESM.docx]

**Appendix 2.** Survey questions and response options

| Questions | Response options |
| --- | --- |
| 1. Which age group do you belong to? | 1 = 29 or younger 2 = 30-39 3 = 40-49 4 = 50-59 5 = 60 or older |
| 2. What is your sex? | 1 = Man 2 = Woman 3 = Other 4 = Prefer not to say |
| 3. Indicate your current professional role: | 1 = Manager (or equivalent role) at a healthcare center (or equivalent) 2 = Manager (or equivalent role) at a nursing home 3 = Physician responsible for a nursing home 4 = Nurse at a nursing home |
| 4. How long have you been working in the professional role indicated above? | 1 = Less than a year 2 = Between 1-2 years 3 = Between 2-5 years 4 = More than five years |
| 5. How long have you been working at your current workplace? | 1 = Less than a year 2 = Between 1-2 years 3 = Between 2-5 years 4 = More than five years 5 = Don't know |
| 6. How do you feel the medical care coordination in eldercare works at the moment? | 4 = Very good 3 = Fairly good 2 = Fairly poor 1 = Very poor |
| 7.1 What is included in medical care coordination at eldercare is clearly stated in agreements or other documents. | 1 = Strongly disagree  2 = 2 3 = 3 4 = 4 5 = 5 6 = 6 7 = Strongly agree 8 = Don't know |
| 7.2 Medical care coordination at eldercare in emergency situations works well. | 1 = Strongly disagree  2 = 2 3 = 3 4 = 4 5 = 5 6 = 6 7 = Strongly agree 8 = Don't know |
| 7.3 The time set aside according to the agreement for medical assistance corresponds to the needs at eldercare. | 1 = Strongly disagree  2 = 2 3 = 3 4 = 4 5 = 5 6 = 6 7 = Strongly agree 8 = Don't know |
| 7.4 The time allocated in practice for medical assistance corresponds to the needs at eldercare. | 1 = Strongly disagree  2 = 2 3 = 3 4 = 4 5 = 5 6 = 6 7 = Strongly agree 8 = Don't know |
| 7.5 The staffing of nurses at eldercare is sufficient for the coordination with the physician to function well. | 1 = Strongly disagree  2 = 2 3 = 3 4 = 4 5 = 5 6 = 6 7 = Strongly agree 8 = Don't know |
| 7.6 It is often the same physician who performs medical interventions at a specific eldercare. | 1 = Strongly disagree  2 = 2 3 = 3 4 = 4 5 = 5 6 = 6 7 = Strongly agree 8 = Don't know |
| 7.7 It is often the same nurse at eldercare who collaborates with the physician in charge. | 1 = Strongly disagree  2 = 2 3 = 3 4 = 4 5 = 5 6 = 6 7 = Strongly agree 8 = Don't know |
| 7.8 Physicians see patients/residents to a sufficient extent. | 1 = Strongly disagree  2 = 2 3 = 3 4 = 4 5 = 5 6 = 6 7 = Strongly agree 8 = Don't know |
| 7.9 It is easy for physicians and nurses at eldercare to get in touch with each other in their daily work. | 1 = Strongly disagree  2 = 2 3 = 3 4 = 4 5 = 5 6 = 6 7 = Strongly agree 8 = Don't know |
| 7.10 Information transfer between healthcare center and eldercare in connection with medical care coordination works well. | 1 = Strongly disagree  2 = 2 3 = 3 4 = 4 5 = 5 6 = 6 7 = Strongly agree 8 = Don't know |
| 7.11 There is a good relationship between physicians and nurses in the daily work. | 1 = Strongly disagree  2 = 2 3 = 3 4 = 4 5 = 5 6 = 6 7 = Strongly agree 8 = Don't know |
| 8. How do you feel that the medical care coordination in eldercare has been affected by the pandemic overall? | 1 = Overall, it has worsened 2 = Overall, it has improved 3 = It has both improved and worsened 4 = It has not been affected at all, it works the same as before 5 = Don't know |
