# Supplementary material for: The impact of Covid-19 on inter-organizational coordination in Swedish eldercare: a mixed methods study
Source: BMC Health Serv Res. 2025 Mar 21;25:416. doi: 10.1186/s12913-025-12576-1 (PMC11927287; doi:10.1186/s12913-025-12576-1)
Supplement: Supplementary file 3 — Supplementary Material 3. [file 12913_2025_12576_MOESM3_ESM.docx]

### Opening questions

Could you briefly share your background and current role within your organization?

For how long have you been employed at your current organization?

What is your role in coordinating medical care between physicians and nurses in your organization?

### Coordination between organizations

Could you describe how medical care coordination is carried out in your organization?

How do you perceive the current state of medical care coordination between physicians and nurses, in nursing homes?

What do you believe contribute to the success/failure of effective medical care coordination in your organization?

### Changes to coordination in the aftermath of Covid-19

To what extent do you perceive that medical care coordination changed in your organization during the pandemic?

(If no change)

Why do you think medical care coordination did not deteriorate?

Why do you think there have been no improvements?

(If change)

Could you describe how you perceive the changes in medical care coordination in your organization during the Covid-19 pandemic?

What do you think contributed to the improvements/deterioration that occurred during the Covid-19 pandemic?

### Sustainable improvements to coordination (if improvements have been made)

How has your organization worked to ensure the sustainability of coordination improvements made over time?

What factors do you believe can contribute to making the improvements in medical care coordination sustainable over time?

What factors do you believe can hinder the sustainability of the improvements in medical care coordination over time?

Is there anything else you would like to add? Is there anything that you feel has not been sufficiently addressed?
