# Supplementary material for: The impact of Covid-19 on inter-organizational coordination in Swedish eldercare: a mixed methods study
Source: BMC Health Serv Res. 2025 Mar 21;25:416. doi: 10.1186/s12913-025-12576-1 (PMC11927287; doi:10.1186/s12913-025-12576-1)
Supplement: Supplementary file 4 — Supplementary Material 4. [file 12913_2025_12576_MOESM4_ESM.docx]

**Appendix 4.** Descriptive statistics of survey questions (7.1-7.11)

| **Descriptive Statistics** | | | | | |
| --- | --- | --- | --- | --- | --- |
|  | **Year** | **Non-response** | **Median** | **Mean** | **SD** |
| 7.1 What is included in medical care coordination at eldercare is clearly stated in agreements or other documents. | 2019 | 17 | 6.000 | 5.529 | 1.365 |
|  | 2022 | 9 | 6.000 | 5.690 | 1.339 |
| 7.2 Medical care coordination at eldercare in emergency situations works well. | 2019 | 17 | 5.000 | 5.053 | 1.705 |
|  | 2022 | 6 | 6.000 | 5.147 | 1.735 |
| 7.3 The time set aside according to the agreement for medical assistance corresponds to the needs at eldercare | 2019 | 17 | 5.000 | 4.843 | 1.963 |
|  | 2022 | 6 | 6.000 | 5.035 | 1.952 |
| 7.4 The time allocated in practice for medical assistance corresponds to the needs at eldercare. | 2019 | 18 | 5.000 | 4.570 | 2.015 |
|  | 2022 | 5 | 5.000 | 4.800 | 2.013 |
| 7.5 The staffing of nurses at eldercare is sufficient for the coordination with the physician to function well. | 2019 | 20 | 6.000 | 4.937 | 1.932 |
|  | 2022 | 5 | 6.000 | 5.077 | 1.926 |
| 7.6 It is often the same physician who performs medical interventions at a specific eldercare. | 2019 | 18 | 6.000 | 5.812 | 1.592 |
|  | 2022 | 6 | 6.000 | 5.856 | 1.597 |
| 7.7 It is often the same nurse at eldercare who collaborates with the physician in charge. | 2019 | 19 | 6.000 | 5.531 | 1.712 |
|  | 2022 | 5 | 6.000 | 5.642 | 1.580 |
| 7.8 Physicians see patients/residents to a sufficient extent. | 2019 | 18 | 5.500 | 4.991 | 1.889 |
|  | 2022 | 9 | 6.000 | 5.033 | 1.851 |
| 7.9 It is easy for physicians and nurses at eldercare to get in touch with each other in their daily work. | 2019 | 16 | 6.000 | 5.385 | 1.705 |
|  | 2022 | 6 | 6.000 | 5.565 | 1.676 |
| 7.10 Information transfer between healthcare center and eldercare in connection with medical care coordination works well. | 2019 | 19 | 6.000 | 5.343 | 1.725 |
|  | 2022 | 6 | 6.000 | 5.409 | 1.693 |
| There is a good relationship between physicians and nurses in the daily work. | 2019 | 19 | 7.000 | 6.185 | 1.298 |
|  | 2022 | 6 | 7.000 | 6.316 | 1.166 |

Appendix 4. Presents a descriptive overview of the survey questions examining the commonly assigned coordination task within medical care coordination, between the years 2019 and 2022.
